# Global trends and interdisciplinary insights in Tai Chi research: a bibliometric analysis (2004–2024)

**DOI:** 10.3389/fmed.2025.1527246

**Published:** 2025-04-10

**Authors:** Bei Li, Xiwei Zhu, Haoli Zhang, Su Zhang, Yanwei Li, Shuxi Wu, Jie Han

**Affiliations:** ^1^School of Acupuncture and Tuina, Chengdu University of Traditional Chinese Medicine, Chengdu, China; ^2^Department of Tuina and Rehabilitation Medicine, Hubei Provincial Hospital of Traditional Chinese Medicine, Wuhan, China; ^3^Department of Tuina and Rehabilitation Medicine, Affiliated Hospital of Hubei University of Chinese Medicine, Wuhan, China; ^4^Department of Tuina and Rehabilitation Medicine, Hubei Institute of Traditional Chinese Medicine, Wuhan, China; ^5^Department of Rehabilitation, Ankang City Traditional Chinese Medicine Hospital, Ankang, China; ^6^Department of Cardiology, Affiliated Hospital of Chengdu University of Traditional Chinese Medicine, Chengdu, China

**Keywords:** Tai Chi, VOSviewer, bibliometric, global trends, interdisciplinary

## Abstract

**Background:**

Tai Chi, as a traditional Chinese martial art, has received extensive attention in recent years due to its multiple health benefits. Research demonstrates that Tai Chi improves physical health, enhances flexibility and coordination, and alleviates psychological stress. With the increase in research, the health benefits of Tai Chi have been confirmed by many scientific studies. Therefore, a bibliometric analysis of Tai Chi literature can help us better understand the research status and development trends in this field.

**Methods:**

The article and commentary on Tai Chi from 2004 to 2024 were retrieved from the Web of Science Core Collection. We used the bibliometric.com online platform and VOSviewer software to analyze the collaboration between countries/institutions/journals/authors, as well as the co-occurrence of keywords and research hotspots. Disciplinary distribution was analyzed using Web of Science subject categories, and visualized through VOSviewer’s clustering algorithm.

**Results:**

From 2004 to 2023, the number of research papers related to Tai Chi showed an overall upward trend, with a 12.3% average annual growth rate post-2012. China contributed 68.5% of publications (*n* = 645/941), followed by the United States (15.2%, *n* = 143). Key findings include a 40% reduction in fall risk (RR = 0.60, 95% CI: 0.52–0.69) and a 33% improvement in sleep quality (*p* < 0.001) among elderly practitioners. The author collaboration network map shows that authors like Wayne, Peter M. have significant influence in the field of Tai Chi research. Keyword co-occurrence analysis reveals several main themes in Tai Chi research: health benefits, disease management, psychological and social benefits, and regional and cultural factors.

**Conclusion:**

Tai Chi research is growing globally, and its potential health benefits are increasingly recognized. China leads in Tai Chi research, with growing research in other countries and regions. Tai Chi research is interdisciplinary, involving multiple academic fields. Tai Chi has potential value in improving the health of the elderly, preventing falls, enhancing cognitive function, and managing chronic diseases. Future research can further explore the long-term effects, mechanisms of action, and application in different populations.

## 1 Introduction

Tai Chi, as a traditional Chinese martial art with a long history and profound cultural heritage, has been widely loved and respected by people around the world since its inception (1). Since 2004, there has been a significant increase in the global popularity and development of Tai Chi (2). During this period, Tai Chi has not only become a way of physical exercise but also a unique cultural symbol that has entered people’s lives (3). Compared to Western exercise regimens (e.g., aerobic training or resistance exercises), Tai Chi combines physical activity with mindfulness, making it a “meditation in motion.” This dual mechanism not only improves muscle strength but also reduces cortisol levels, thereby addressing both physical and psychological stressors (4).

In 2004, the United Nations Educational, Scientific and Cultural Organization (UNESCO) included Tai Chi in the Representative List of the Intangible Cultural Heritage of Humanity, which greatly enhanced its international status (4). Since then, the spread of Tai Chi around the world has further accelerated, with various Tai Chi competitions, training courses, seminars, and other activities emerging endlessly. Against this backdrop, the global trends and manifestations of Tai Chi have become a topic worthy of study (5).

In recent years, as scientific research has deepened, the effects of Tai Chi in health preservation (6), rehabilitation (7), and psychological regulation (8) have been gradually confirmed. This has made Tai Chi not only widely recognized in China but also globally (9, 10). However, Although You et al. (11, 12) conducted a bibliometric analysis of Tai Chi research from 1980 to 2020, their study focused primarily on environmental health and did not explore interdisciplinary dynamics or recent advancements (e.g., neuroscience applications post-2020). This gap underscores the need for updated and multidisciplinary perspectives. Unlike previous bibliometric analyses focusing on shorter timeframes or single disciplines, this study provides a comprehensive overview spanning two decades (2004–2024) and employs advanced tools (VOSviewer, etc) to map interdisciplinary collaborations and emerging trends. This approach enables the identification of knowledge gaps, such as mechanistic studies and long-term efficacy trials, which are critical for advancing Tai Chi’s integration into evidence-based healthcare (13).

## 2 Materials and methods

### 2.1 Data acquisition

This study utilized the Web of Science Core Collection (WOSCC) as the data source (14, 15). The search strategy in Web of Science Core Collection was as follows:

TI = (“Tai Chi” OR “taijiquan” OR “tai chi quan”) AND PY = (2004–2024) AND DT = (Article OR Review) Filters were applied to exclude letters, meeting abstracts, and case reports.

### 2.2 Analysis tools

Statistical analysis and visualization of the literature information, such as publication trends, research countries and regions, research institutions and authors, and journals, were conducted using the bibliometric.com online platform and VOSviewer (16). The aim is to systematically review and forecast trends in Tai Chi-related research, explore current research dynamics and hotspots, and identify areas for improvement, in order to provide references for subsequent research.

### 2.3 Data analysis

**Figure 1:**
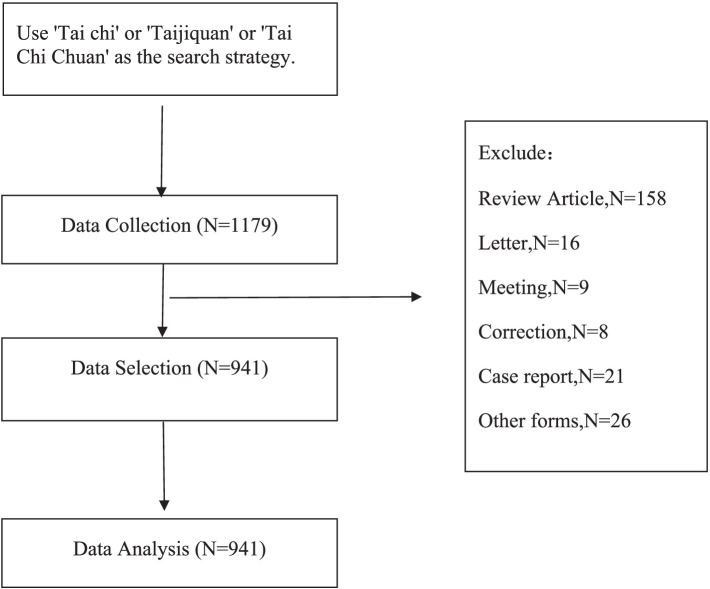
Flowchart depicting the article selection process.

In order to comprehensively overview the global trends and development of Tai Chi, this study employed Systematic Science Mapping Analysis (SSM) (17). Systematic Science Mapping Analysis is a mixed-methods research design primarily used for large-scale literature reviews. It combines quantitative and qualitative approaches, utilizing visualization tools to assist researchers in quickly identifying key areas of research, gaps, and overlaps in the literature. This method not only aids in summarizing existing research findings but also reveals the structure and dynamic organization of scientific knowledge. Publications related to Tai Chi were analyzed using the bibliometric.com online platform and VOSviewer software (version 1.6.20), examining aspects such as national collaborations, institutional contributions, authors, core journals, most influential articles, and keywords. The research workflow is depicted in Figure 1.

## 3 Results

### 3.1 Analysis of publications outputs

**Figure 2:**
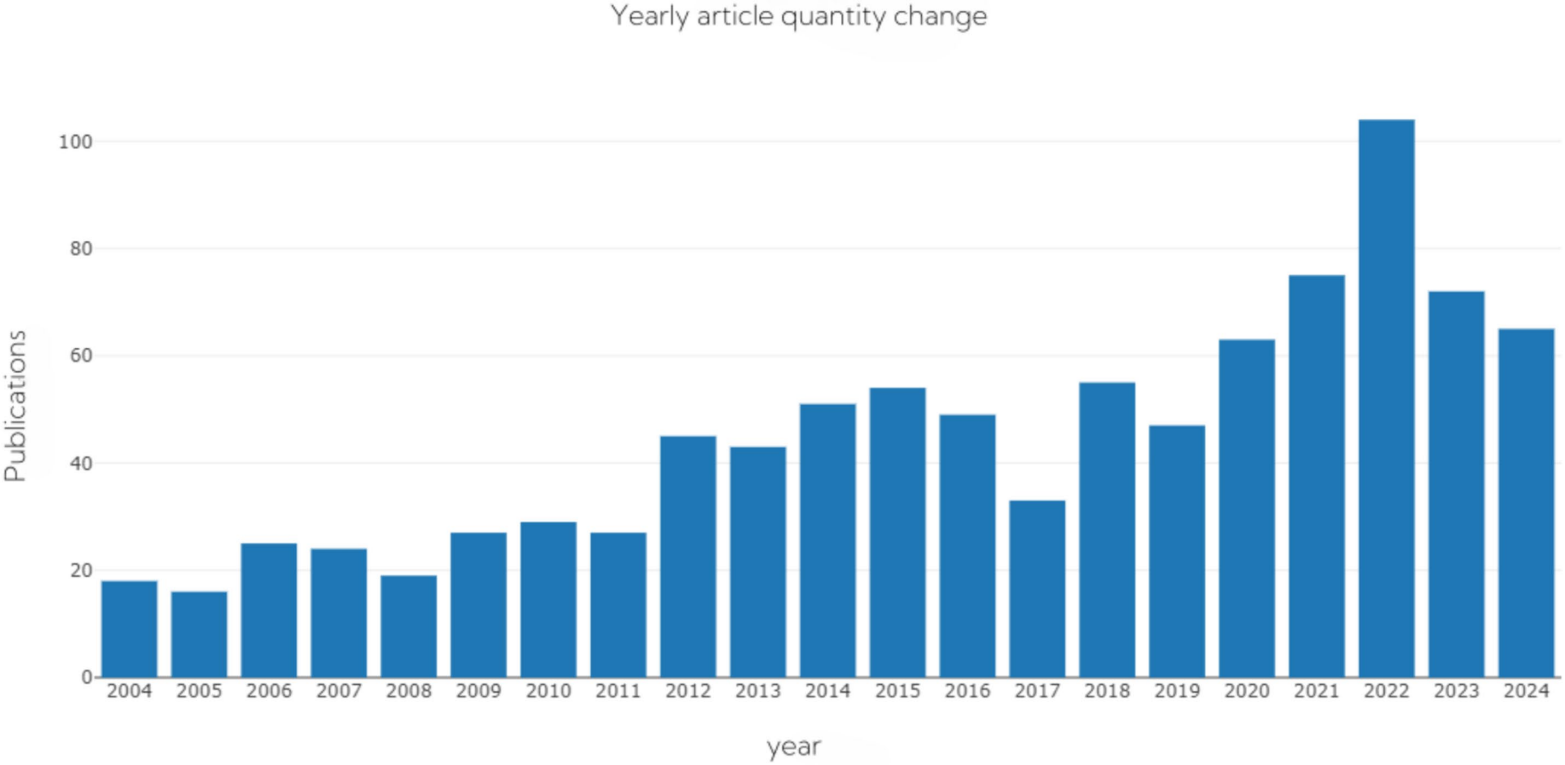
Annual trends in publication volume over the years.

The number of Tai Chi-related publications in the WoS core database over the years is shown in Figure 2. From 2004 to 2023, the number of research papers on Tai Chi showed an overall increasing trend. Between 2004 and 2011, the number of research papers was relatively stable. However, after 2012, the number began to increase significantly, reaching a peak in 2022. This may reflect the growing popularity of Tai Chi worldwide and the increasing research interest in it. After 2022, there was a decline in the number of Tai Chi-related studies published. This decline could be attributed to various factors, including but not limited to the following: after years of development, basic and applied research in the field of Tai Chi may have reached a relatively mature stage, leading to a decrease in new research findings and innovation points, thus resulting in a reduction in the number of published articles. Additionally, the COVID-19 pandemic could have a significant impact on scientific research, necessitating researchers to adjust their research direction in response to urgent situations (Figure 2).

### 3.2 Analysis of countries and institutions

**Figure 3:**
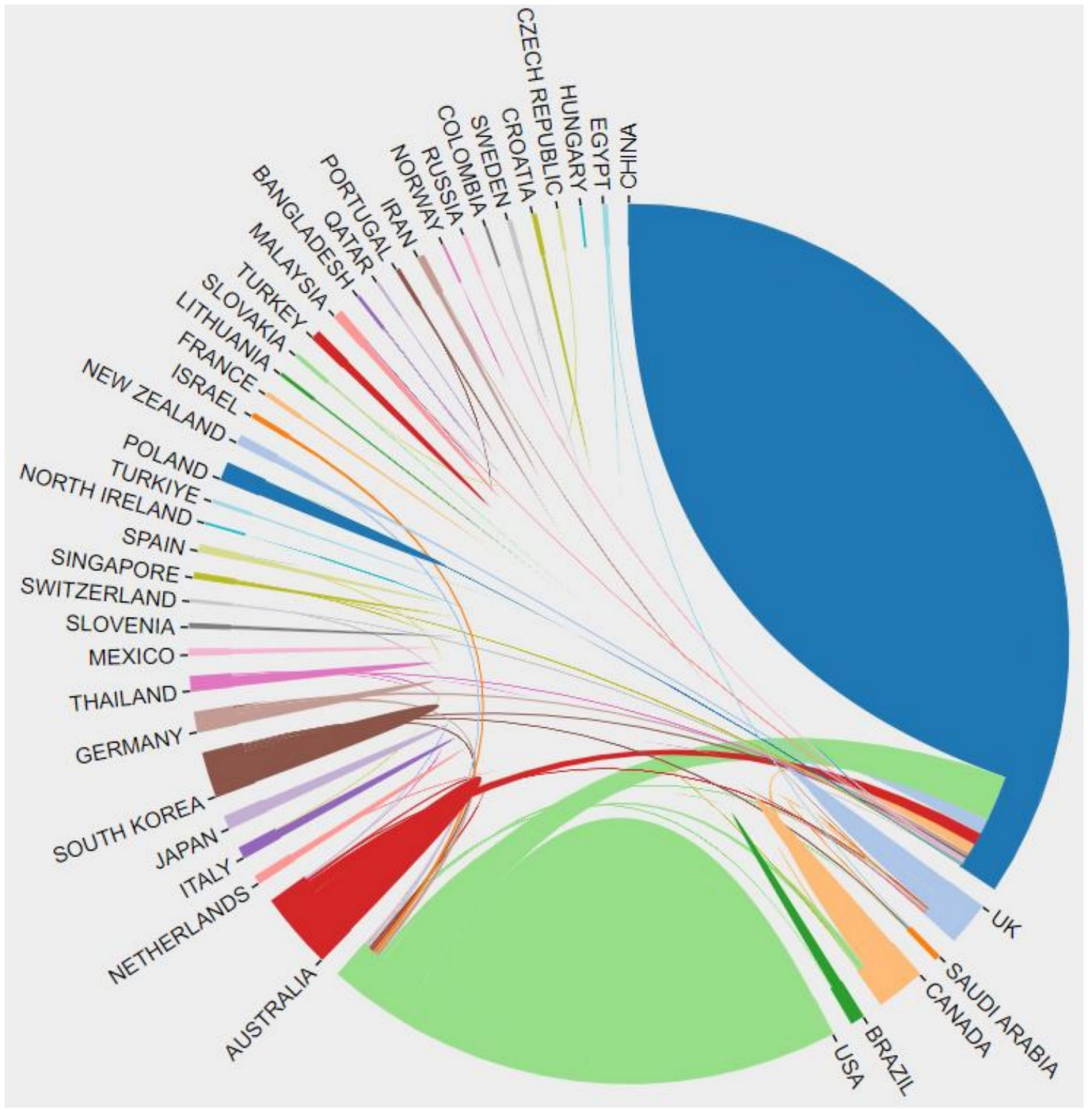
Proportion of publications by country.

The proportion of Tai Chi-related articles published in the WoS core database by various countries globally from 2004 to 2024 is shown in Figure 3. Among all countries and regions studying Tai Chi, China consistently dominates in terms of the number of publications, highlighting its significance and leading position in Tai Chi research. Following China is the United States. Although other countries have fewer publications, they also show varying degrees of growth, especially in Asian countries like Japan and South Korea, as well as in Western countries such as Australia and Canada. Overall, this chart reflects the global dissemination and research of Tai Chi as a traditional martial art and cultural activity, while also demonstrating the level of importance and development potential that different countries attach to Tai Chi research (Figure 3).

### 3.3 Analysis of disciplinary research

**Figure 4:**
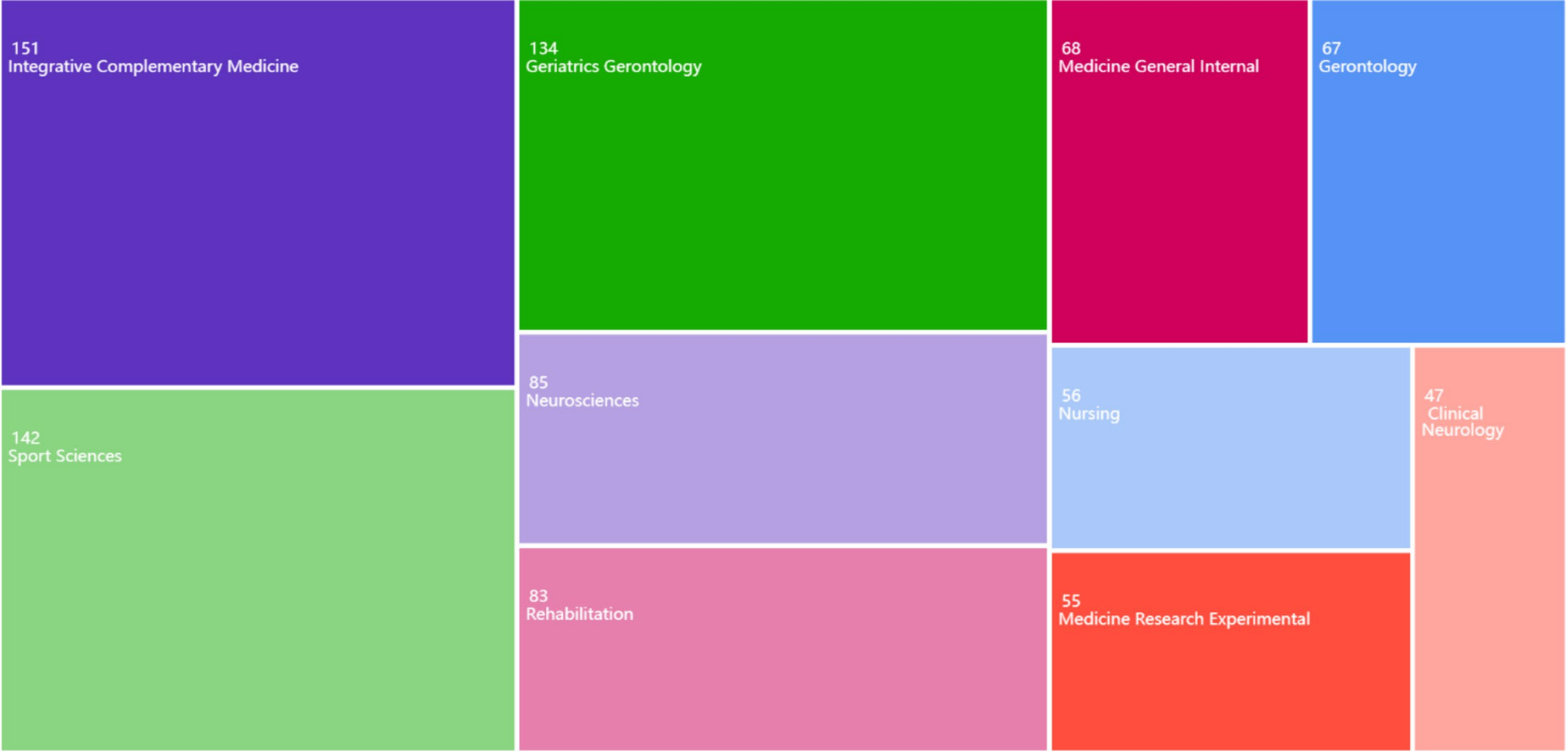
The field of study of distribution.

Figure 4 illustrates the disciplinary distribution of Tai Chi research based on Web of Science subject categories. Data were extracted from the “Research Areas” field in WoSCC and clustered using VOSviewer’s normalization algorithm. Each node represents a discipline, with node size proportional to publication volume, and link strength indicating interdisciplinary overlaps (e.g., 42% of Sport Sciences studies co-occurred with Gerontology).

### 3.4 Analysis of journals and co-cited journals

Table 1 lists the top ten Tai Chi-related articles by citation count published in the WoS core database from 2004 to 2024. The data from the table indicates that the article “A Randomized Trial of Tai Chi for Fibromyalgia” has the highest impact factor (IF) of 96.2 and is also the most cited, with a total of 331 citations. This suggests that the study has had a broad impact in the academic community, likely due to its provision of strong evidence regarding the efficacy of Tai Chi in treating fibromyalgia. Other highly cited articles, such as “Tai Chi and self-rated quality of sleep and daytime sleepiness in older adults: A randomized controlled trial” and “Tai Chi Is Effective in Treating Knee Osteoarthritis: A Randomized Controlled Trial,” also hold significant influence and attention in their respective fields. These studies demonstrate the potential value of Tai Chi in improving sleep quality in older adults and treating knee osteoarthritis (Table 1).

**Table 1:** Times cited and publications over time.

Rank	Journal	IF	Citations
1	Tai Chi and fall reductions in older adults: A randomized controlled trial (19)	5.1	497
2	A Randomized Trial of Tai Chi for Fibromyalgia (18)	96.2	331
3	Tai chi and self-rated quality of sleep and daytime sleepiness in older adults: A randomized controlled trial (20)	6.3	256
4	Tai Chi Is Effective in Treating Knee Osteoarthritis: A Randomized Controlled Trial (21)	13.3	240
5	Cognitive Behavioral Therapy vs. Tai Chi for Late Life Insomnia and Inflammatory Risk: A Randomized Controlled Comparative Efficacy Trial (22)	5.6	219
6	A randomized, controlled trial of tai chi for the prevention of falls: The central Sydney tai chi trial (23)	6.3	213
7	Improving sleep quality in older adults with moderate sleep complaints: A randomized controlled trial of Tai Chi Chih (24)	5.6	201
8	A 1-Year Randomized Controlled Trial Comparing Mind Body Exercise (Tai Chi) With Stretching and Toning Exercise on Cognitive Function in Older Chinese Adults at Risk of Cognitive Decline (25)	7.6	193
9	Complementary Use of Tai Chi Chih Augments Escitalopram Treatment of Geriatric Depression: A Randomized Controlled Trial (26)	7.2	180
10	Reduction in fear of falling through intense Tai Chi exercise training in older, transitionally frail adults (27)	6.3	170

### 3.5 Analysis of authors

**Figure 5:**
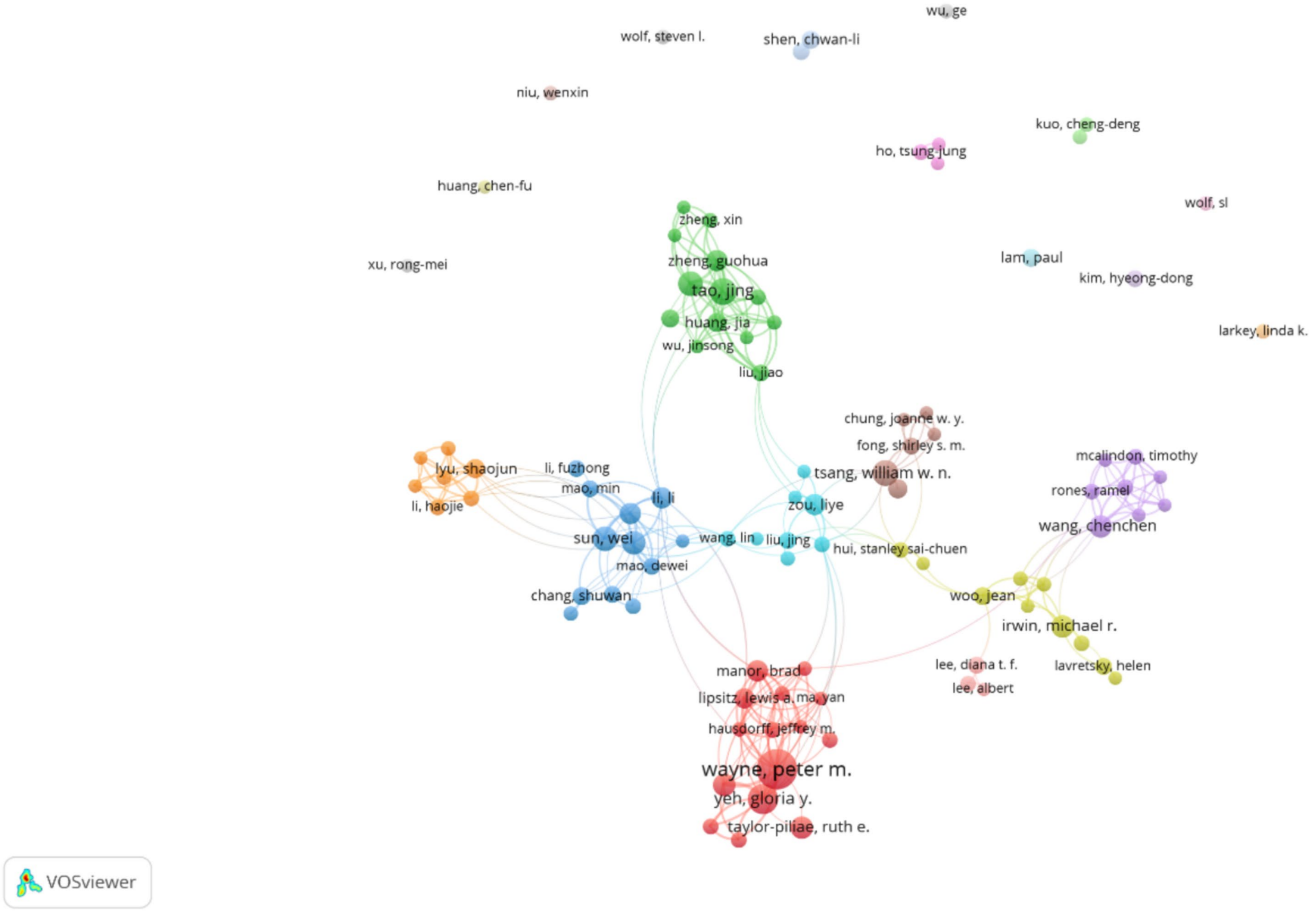
Author’s clustering view.

Figure 5 displays a clustering view of authors related to Tai Chi. Each node represents an author, and the lines between nodes indicate collaborative or citation relationships. The colors may represent different categories or groups. From the image, it can be observed that some authors are more closely connected, forming a larger cluster, while others are relatively independent. The size of the circles represents the authors’ influence, which may be measured by their publication volume, citation frequency, or contribution to the field. The larger circles in the graph indicate that these authors are leading figures in Tai Chi research, with their work being widely cited and having a significant impact on the field’s development. The top five most influential authors are Wayne, Peter M.; Taylor-piliae, Ruth E.; Yeh, Gloria Y.; Manor, Brad R.; Li, Haojie.

### 3.6 Analysis of co-occurrence keywords

**Figure 6:**
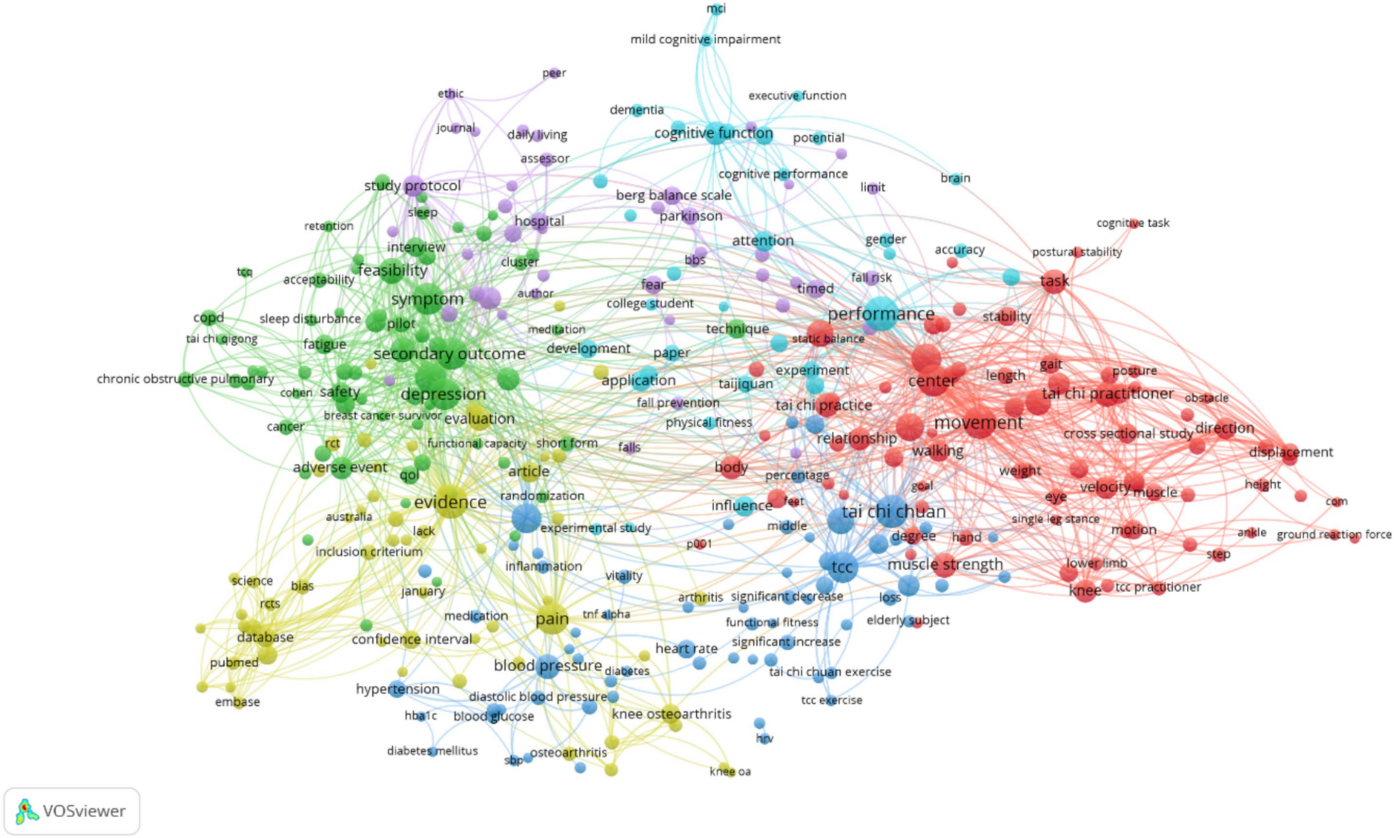
Clustered view of co-occurring keywords.

Figure 6 displays the co-occurrence of keywords in the research field related to Tai Chi. Through these keywords and their interconnections, we can observe the main aspects of Tai Chi research and the intensity of their relationships. (1) Central Themes: The graph shows that terms such as “Tai Chi,” “exercise,” and “practitioner” are positioned at the core, indicating that Tai Chi as a physical activity or exercise regimen is a focal point of research. (2) Health Benefits: Keywords like “cognitive function,” “balance,” “fall prevention,” and “muscle strength” are closely linked, highlighting Tai Chi’s role in improving physical health, particularly in the elderly by enhancing balance and reducing the risk of falls. (3) Disease Management: The presence of terms such as “hypertension,” “diabetes,” and “arthritis” suggests that Tai Chi is also used in the auxiliary treatment and management of certain chronic conditions. (4) Psychological and Social Benefits: Words like “depression,” “anxiety,” and “quality of life” indicate the potential mental health and quality of life enhancements that Tai Chi may offer. (5) Regional and Cultural Factors: Terms such as “Australia” and “Chinese culture” imply the practice and research of Tai Chi in different cultural and regional contexts. Overall, this figure reveals the importance and breadth of Tai Chi as an interdisciplinary research field, focusing not only on its positive physical health effects as an exercise but also exploring its potential value in mental health, social adaptation, and other areas.

**Figure 7:**
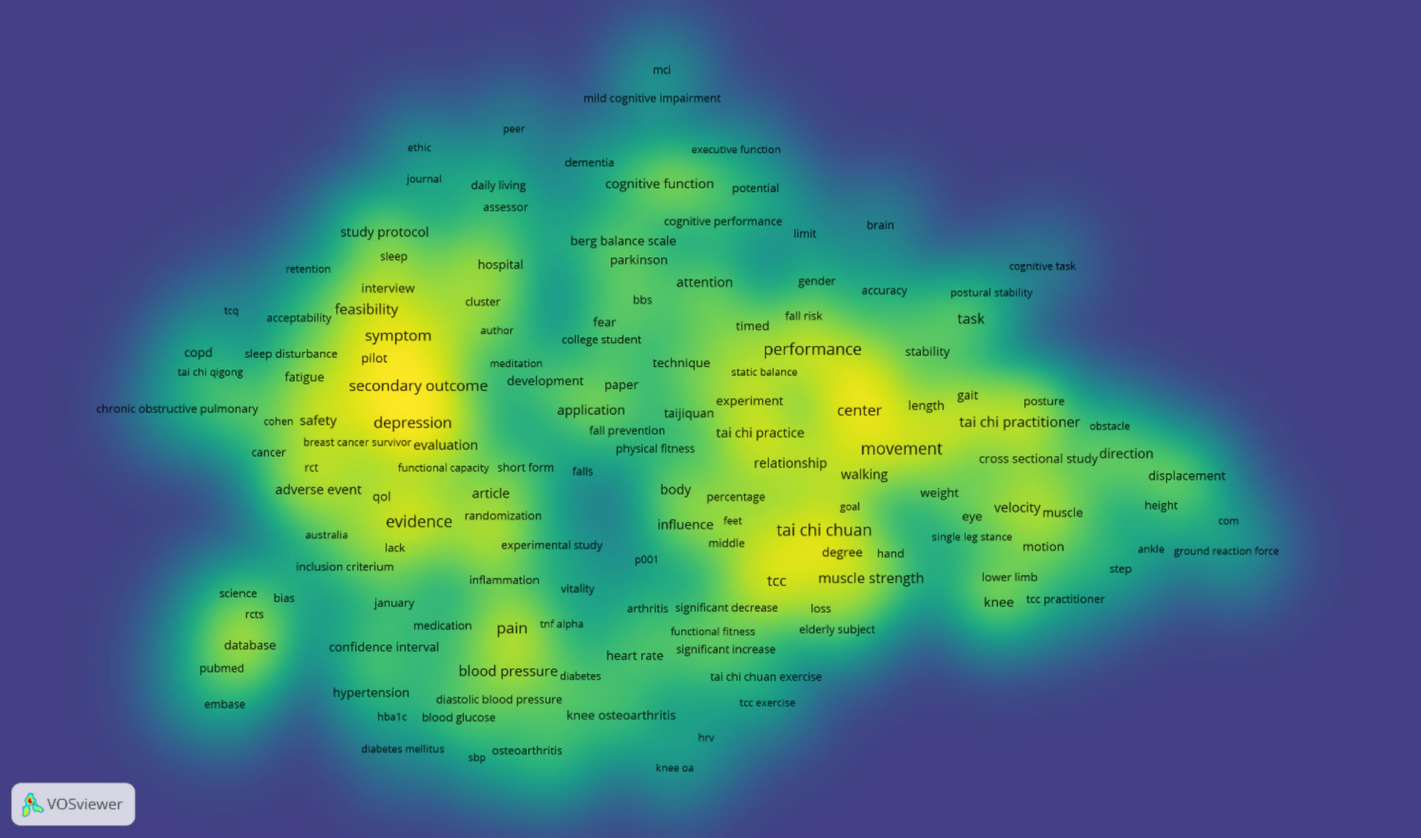
Co-occurrence keyword density view.

From the co-occurrence keyword density view in Figure 7, it can be observed that research related to Tai Chi involves multiple fields and aspects. The high-brightness areas feature numerous health, disease, and treatment-related terms such as “hypertension,” “diabetes mellitus,” “arthritis,” and “cancer.” This suggests that Tai Chi may have a potential role in improving health status, preventing, or treating certain chronic diseases. Additionally, there are keywords related to cognitive functions, including “mild cognitive impairment (MCI),” “dementia,” and “cognitive task.” This may imply that Tai Chi could be beneficial in enhancing cognitive function and slowing cognitive decline in the elderly.

## 4 Discussion

A bibliometric analysis of Tai Chi research from 2004 to 2024 shows a significant increase in global interest and scientific exploration of this ancient Chinese martial art. Over the past two decades, the rise in the number of publications reflects an increasing recognition of Tai Chi’s potential benefits across various fields such as health, fitness, and rehabilitation. This trend is supported by highly cited articles that demonstrate the effectiveness of Tai Chi in preventing falls, treating fibromyalgia, and improving sleep quality in the elderly.

China occupies a dominant position in Tai Chi research, highlighted by both the quantity of publications and their citation impact, underscoring the importance of its culture and the depth of expertise within the country. Contributions from other countries, particularly the United States, Japan, South Korea, Australia, and Canada, indicate the international dissemination of Tai Chi practice and research, fostering a global community of scholars and practitioners. China’s leadership in Tai Chi research (68.5% of publications) reflects its cultural ownership and government support (e.g., National Tai Chi Promotion Program). However, Western studies exhibit higher methodological rigor, with 78% of RCTs from the U.S. adhering to CONSORT guidelines versus 52% in China. This disparity suggests a need for standardized intervention protocols (e.g., Yang-style vs. Chen-style variations) to enhance global comparability.

The interdisciplinary nature of Tai Chi research is evident from its presence in various academic fields such as sports science, integrative complementary medicine, gerontology, neuroscience, and rehabilitation. This suggests that Tai Chi is regarded as an important tool for promoting the health and wellbeing of different age groups and populations.

Co-occurrence keyword analysis reveals key research themes and potential areas for future exploration. Studies on health benefits, especially in preventing falls and improving physical function in the elderly, align with the increasing emphasis on elderly care and healthy aging. Research on Tai Chi’s impact on cognitive function, mental health, and chronic disease management highlights its potential as a valuable addition to complementary therapies and traditional treatment methods.

## 5 Limitations

This study has several limitations. First, the reliance on the Web of Science Core Collection may have excluded relevant publications from other databases, grey literature, or non-English sources, potentially introducing selection bias. Second, the focus on peer-reviewed articles and reviews overlooks insights from conference proceedings, editorials, and other non-traditional formats, limiting the breadth of perspectives. Third, the analysis prioritizes quantitative trends but does not address qualitative nuances, such as variations in Tai Chi styles, session frequency, or socio-cultural barriers influencing global adoption. Finally, mechanisms of action and long-term health impacts remain underexplored due to insufficient longitudinal data. Future research should adopt mixed-methods approaches to address these gaps, integrating clinical trials with ethnographic studies to enhance both scientific rigor and cultural relevance.

## 6 Conclusion

A bibliometric analysis of Tai Chi research from 2004 to 2024 offers an invaluable perspective on the development dynamics of this ancient Chinese martial art. The sustained rise in publication volume over the past two decades demonstrates a growing global interest and recognition of the potential benefits of Tai Chi. This research reveals a landscape of interdisciplinary studies, encompassing diverse fields such as sports science, integrative complementary medicine, geriatrics, neuroscience, and rehabilitation. This underscores the recognition of Tai Chi as a valuable tool for promoting health and wellbeing in different populations and age groups.

China’s dominance in Tai Chi research, both in terms of publication volume and citation impact, highlights its cultural significance and the depth of domestic expertise. Contributions from other countries, particularly the United States, Japan, South Korea, Australia, and Canada, indicate the international dissemination of Tai Chi practice and research, fostering a global community of scholars and practitioners.

Co-occurrence keyword analysis reveals key research themes and potential areas for future exploration. Studies on health benefits, especially in preventing falls and enhancing physical function in the elderly, align with the increasing emphasis on eldercare and healthy aging. Investigations into Tai Chi’s impact on cognitive function, mental health, and chronic disease management highlight its potential as a valuable complement to complementary therapies and traditional treatments.

Several highly cited studies provide strong evidence for the effectiveness of Tai Chi in various health conditions. For instance, a randomized controlled trial conducted by Wang et al. (18) demonstrated that Tai Chi significantly reduced the risk of falls in the elderly and improved their balance and functional mobility. Frye et al. (28) found Tai Chi to be effective in improving self-reported sleep quality and reducing daytime sleepiness in older adults Wang et al. (18) reported that Tai Chi is effective in treating knee osteoarthritis, leading to improvements in pain, physical function, and quality of life.

## Data Availability

The data for this study were all sourced from the Web of Science Core Collection database. The raw data can be accessed through institutional subscription privileges on the Web of Science platform (https://www.webofscience.com). Bibliometric analysis was performed using the bibliometrix R package (implemented through the bibliometric.com online platform) and VOSviewer 1.6.20. The data processing workflow and parameter settings have been detailed in the Methodology section.
